# Traumatic brachial plexus injuries: a study of 665 cases from multicenter services in Guangxi, China (2017–2022)

**DOI:** 10.3389/fneur.2026.1906794

**Published:** 2026-07-27

**Authors:** Guang-Yao Li, Jie Luo, Yi-Jin Gao, Jing-Wei Wang, Lang Cai, Ke Sha

**Affiliations:** 1 Department of Orthopedic Trauma and Hand Surgery, The First Affiliated Hospital of Guangxi Medical University, Nanning, Guangxi, China; 2 Department of Orthopedics, The Second Nanning People’s Hospital, Nanning, Guangxi, China; 3 Department of Orthopedics, Wuming Hospital of Guangxi Medical University, Nanning, Guangxi, China

**Keywords:** brachial plexus injury, e-bike, epidemiology, nerve reconstruction, treatment

## Abstract

**Background:**

Traumatic brachial plexus injury (BPI) is a severe condition whose epidemiological characteristics exhibit regional and temporal variation, potentially affecting its diagnosis and treatment.

**Methods:**

A retrospective multicenter study was conducted across a regional healthcare network comprising 16 tertiary and 3 secondary hospitals. A total of 683 patients with traumatic BPI treated between January 2017 and December 2022 were enrolled, of whom 665 were included in the final analysis. Demographic and clinical data were extracted from the electronic medical record system. Limb function was assessed at final follow-up using the Louisiana State University Medical Center (LSUMC) grading system, and statistical analyses were performed using SPSS version 22.0.

**Results:**

Of the 665 patients, 480 were male and 185 were female, with a mean age of 41.65 years. The proportion of female patients increased significantly from 15.6% in 2017 to 34.1% in 2022. Among traffic-related injuries (45.86%), e-bike accidents were the most common mechanism (27.82%), surpassing motorcycle accidents (13.23%). Closed injuries accounted for 90.98% of cases, Fractures were present in 51.43% of patients, with clavicular fractures having the highest surgical rate (98.82%). Surgery within 3 months achieved a recovery rate of 78.5%, which was significantly higher than that of delayed surgery (*p* < 0.001). Among surgical patients, isolated infraclavicular injuries had the highest recovery rate (82.35%), while C5–T1 injuries had the lowest (49.33%). Neurolysis yielded the highest recovery rate (79.17%).

**Conclusion:**

The epidemiology of BPI in Guangxi has shifted markedly, with notable age differences between male and female patients and e-bike accidents emerging as the dominant mechanism. Early intervention and individualized surgery remain critical determinants of favorable outcomes.

## Introduction

The brachial plexus (BP) is formed by the anterior rami of C5–T1 and provides sensory and motor innervation to the upper limb. Traumatic brachial plexus injury (BPI) often leads to significant and long-lasting limb dysfunction. This imposes substantial occupational and psychological burdens on affected individuals (1, 2). Despite advances in microsurgery that have improved BPI outcomes (3), the prognosis for severe injuries like total BPI remains poor (4).

The epidemiological characteristics of BPI are shaped by regional and temporal variation. As early as 1985, Narakas (5) reported that BPI predominantly affects young males, with traffic accidents constituting the leading cause of injury. However, recent studies from other regions have shown new changes in this epidemiological pattern. In the United States, with the dissemination of surgical techniques and knowledge, the annual surgical incidence of BPI rose from 1.26 to 1.91 per 100,000 person-years between 2009 and 2019. While young men remained the most affected group, the number of elderly and female patients has increased (6). Furthermore, a 32-year study in England and Wales documented a median patient age of 41 years, and 77% were male. Male patients younger than 65 years were significantly more likely to sustain BPI secondary to traffic accidents, whereas female patients aged 65 years and older faced higher risk from falls of less than 2 m (7). Whether epidemiological changes have occurred in our region remains unknown. To clarify the actual situation of BPI, a retrospective study was conducted examining the demographic data, clinical treatment and follow-up outcomes of 683 consecutive patients with BPI.

## Methods

### Participants

This was a multicenter retrospective cross-sectional study, which was approved by the Ethics Committee (Approval No. 2016-E0023). A total of 683 patients with traumatic BPI were enrolled from our regional healthcare network—comprising 16 tertiary hospitals and 3 secondary hospitals—between January 2017 and December 2022. Eligible patients had a confirmed trauma history with clinical and electrophysiological findings consistent with BPI. Patients with incomplete records were excluded.

### Patient data

All data were extracted from the electronic medical record system: (1) demographic data, including sex, age, educational level, occupation, and household registration; (2) clinical data, covering medical history, injury site, injury severity, concomitant injuries; (3) surgical information, containing the time from injury to surgery, surgical procedures, and intraoperative findings. For classification purposes, BPI was categorized into supraclavicular and infraclavicular types.

### Follow-up survey

Patients were followed up through outpatient visits or telephone, with a minimum duration of 24 months. Of the 683 enrolled patients, 18 were excluded: 2 died, 6 declined further participation, and 10 were lost to follow-up due to changes in contact information. The remaining 665 patients (97.36%) were included in the final analysis.

Upper limb function was assessed using the Louisiana State University Medical Center (LSUMC) grading system (8), which ranges from Grade 0 (no palpable contraction) to Grade 5 (full strength against resistance). Successful recovery was defined as improvement in composite muscle strength for specified joint movement direction to Grade 3 or higher.

### Statistical analysis

All statistical analyses were performed using SPSS version 22.0 (IBM Corp., Armonk, NY, USA). Categorical variables were reported as frequencies and percentages, and continuous variables were expressed as means ± standard deviations (SD). Group comparisons for categorical variables were performed using Pearson’s chi-square test or Fisher’s exact test, as appropriate, with *post-hoc* Bonferroni correction applied when the overall comparison was significant. A two-sided *p* value < 0.05 was considered statistically significant.

## Results

### Epidemiology

**Figure 1:**
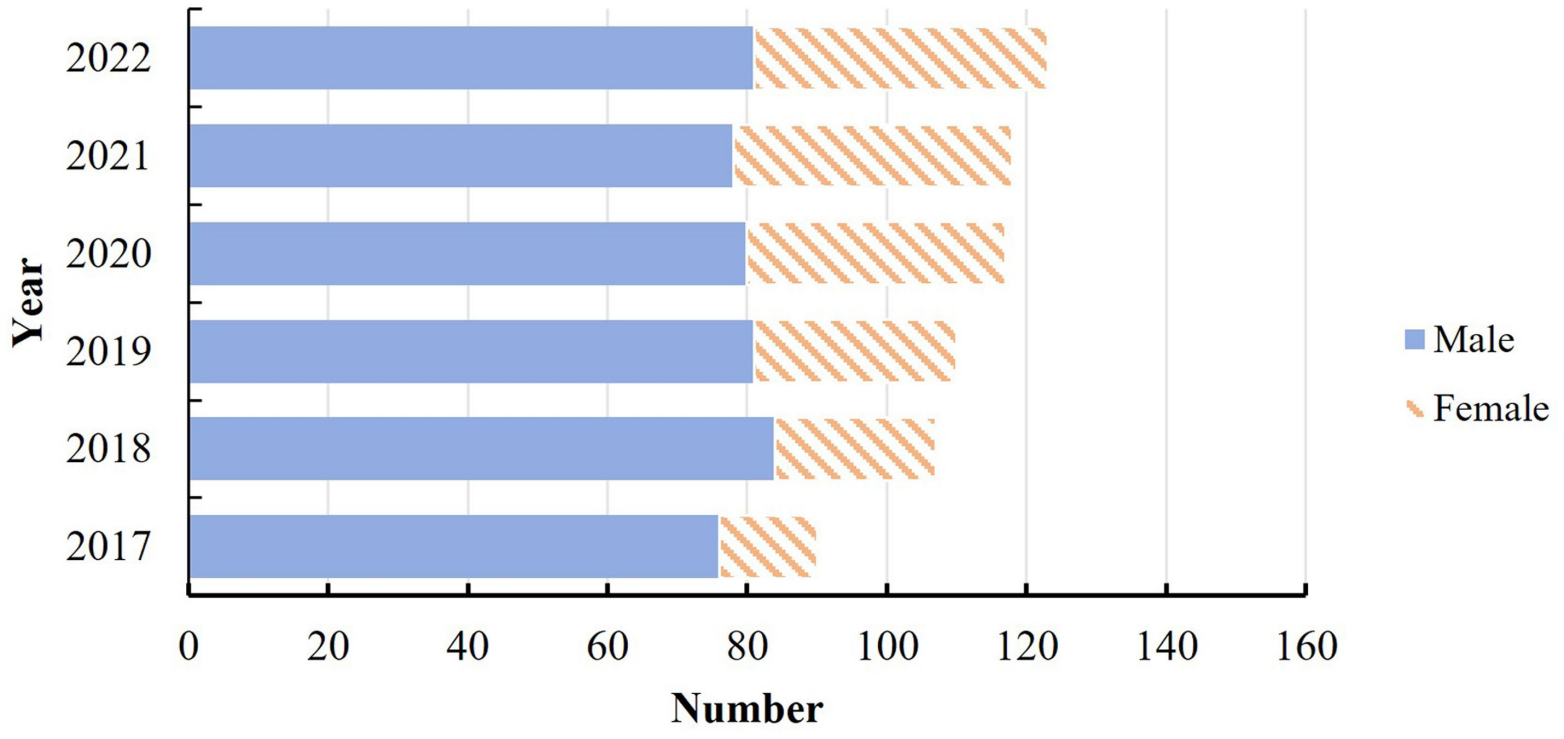
Annual number of patients with BPI.

**Figure 2:**
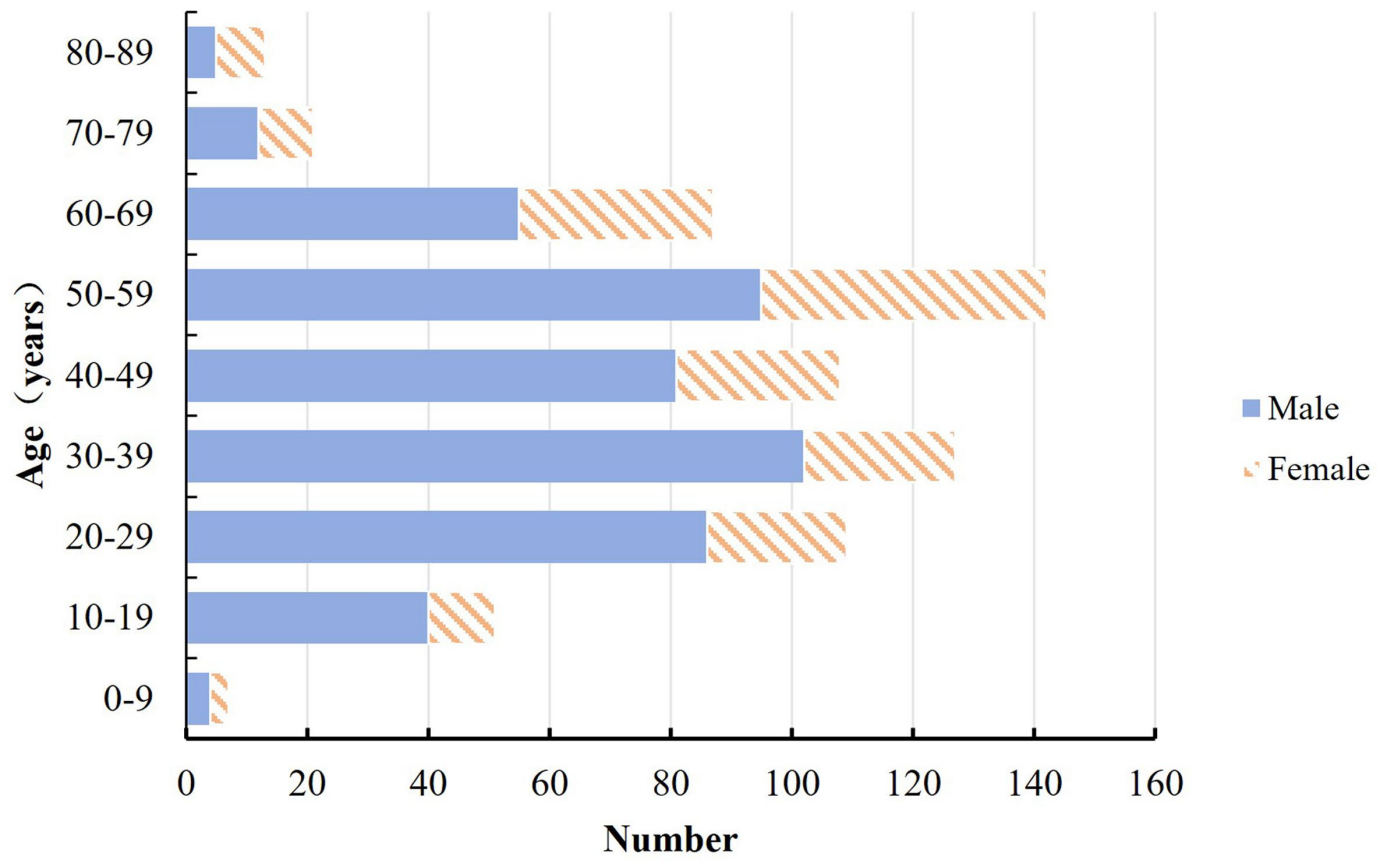
Histogram of the distribution of patient’ ages.

This study comprised 480 males (72.18%) and 185 females (27.82%), with a male-to-female ratio of 2.59:1. The proportion of female patients increased from 15.6% in 2017 to 34.1% in 2022 (Figure 1). The majority of BPI cases were closed (90.98%) and unilateral (98.50%), with left- and right-sided injuries accounting for 50.83 and 47.67%, respectively. The mean age was 41.65 ± 17.37 (range, 5–89 years). Among all age groups, the 50–59 age group had the highest proportion of patients (21.35%, Figure 2).

Most patients held rural household registration (78.95%). Additionally, 12.78% were referred from neighboring provinces. Farmers constituted the largest occupational group (39.10%), followed by workers (24.96%) and the unemployed (10.23%). The majority of patients reported low income (72.93%), and 86.47% had an educational level of high school or below. Road traffic accidents were the leading injury mechanism (45.86%, Table 1), among which electric bike (e-bike) accidents (27.82%) were more common than motorcycle accidents (13.23%). Falls from less than 2 m (17.44%), industrial injuries (14.29%), and falls from greater than 2 m (12.48%) followed in frequency.

**Table 1:** List and frequency of the injury mechanism of BPI.

ReferencesMechanism	Dubuisson and Kline (19) 2002 (*n* = 100)	Kandenwein et al. (14) 2005 (*n* = 134)	Faglioni et al. (15) 2014 (*n* = 406)	Li et al. (9) 2019 (*n* = 510)	Zaidman et al. (2) 2024 (*n* = 64)	Warwick and Hems (48), 2024 (*n* = 425)	Li et al. 2026 (*n* = 665)
Traffic accidents							
Motorcycle	27.00%	50.75%	79.00%	46.27%	32.80%	11.76%	13.23%
Bicycle	4.00%	5.97%	3.20%	—	—	4.47%	—
Electric bike	—	—	—	9.22%	—	—	27.82%
Car	21.00%	14.18%	3.80%	4.71%	20.30%	6.59%	3.61%
Other	8.00%	10.45%	3.80%	4.51%	6.30%	1.41%	1.20%
Falls >2 m	2.00%	2.99%	2.20%	16.08%	7.80%	11.29%	12.48%
Falls <2 m	5.00%	—	—	—	—	37.65%	17.44%
Industrial injury	3.00%	—	—	13.72%	3.10%	2.59%	14.29%
Sharp injury	6.00%	2.24%	—	3.14%	4.70%	—	2.11%
Long-time traction	—	—	—	—	—	10.12%	4.51%
Iatrogenic injury	9.00%	—	—	1.37%	—	6.12%	1.80%
Gunshot wound	8.00%	5.97%	4.10%	0.98%	17.20%	—	—
Sports-related	3.00%	—	—	—	—	3.29%	—
Others	4.00%	7.46%	3.90%	—	7.80%	4.71%	1.50%

The data is issued from 6 previously published series of the literature and from the present series.

### Preoperative condition

Although 96.39% of patients presented within 24 h of injury, only 58.65% received a correct diagnosis of BPI at initial presentation. Fractures were the most common concomitant injury (*n* = 342, 51.43%), followed by shoulder dislocation (n = 135, 20.30%) and thoracic injury (*n* = 69, 10.38%; Table 2). Among patients with fractures, the humerus was the most frequently affected site (*n* = 183, 53.51%), despite clavicle fractures having the highest surgical rate (98.82%, Table 3). Associated neurological signs included Horner syndrome (*n* = 57), trapezius paralysis (*n* = 32), hemidiaphragmatic paralysis (*n* = 9), and winged scapula (*n* = 6).

**Table 2:** Concomitant injuries in patients with BPI.

Injuries	No. of patients	Percentage (%)
Fracture	342	51.43
Shoulder dislocation	135	20.30
Thoracic injury (excluding fractures)	69	10.38
Brain or spinal cord injury	61	9.17
Muscle injury or ischemic muscle spasm	33	4.96
Great vascular injury	21	3.16
Abdominal trauma	10	1.50

**Table 3:** Distribution of fractures associated with BPI.

Fracture sites	No. of patients	Percentage (%)	Surgical rate (%)
Humerus	183	27.52	71.04
Ribs	88	13.23	59.09
Scapula	86	12.93	37.21
Clavicle	85	12.78	98.82
Bones of the forearm and hand	72	10.83	90.28
Spine	65	9.77	49.23
Bones of the lower limb	50	7.52	92.00
Skull	41	6.17	14.63
Pelvis	11	1.65	90.91

Supraclavicular injury was the most frequent type (65.56%), predominantly involving nerve root and trunk lesions (Table 4). Cord and branch injuries were significantly more prevalent in the non-surgical group than in the surgical group (41.72% vs. 20.80%), whereas combined supraclavicular and infraclavicular injuries were more common in the surgical group.

**Table 4:** Distribution of injury sites by treatment group.

Injury site	Non-surgical group (*n* = 338)	Surgical group (*n* = 327)	Total (*n* = 665)	*p*-value
Root/Trunk	194 (57.40%)	242 (74.01%)	436 (65.56%)	*p* < 0.001
Cord/Branch	141 (41.72%)	68 (20.80%)	209 (31.43%)	
Supraclavicular and infraclavicular	3 (0.89%)	17 (5.20%)	20 (3.01%)	

### Treatment

Of the 665 patients, 338 received non-surgical management and 327 underwent surgery. Among surgical patients, 195 (59.63%) underwent surgery within 3 months of injury, 70 (21.41%) between 3 and 6 months, and 62 (18.96%) beyond 6 months. Intraoperative findings were summarized in Table 5; C5–C6 injury was the most common pattern (27.22%), followed by C5–T1 (22.94%) and C5–C7 (19.27%). Common pathological findings included scar tissue, adhesions, nerve contusions, and edema. Of the 327 surgical patients, 175 had injuries involving the nerve roots, trunks, cords, or branches, of whom 41 presented with total root avulsion (RA). A total of 315 nerve roots were avulsed, with C5 (*n* = 75), C6 (*n* = 74), and C7 (*n* = 69) being the most frequently involved root.

**Table 5:** The distribution of BPI in surgical patients (*N* = 327).

Injury pattern	No. of patients	Percentage (%)
C5–C6	89	27.22
C5–C7	63	19.27
C7–T1	6	1.83
C8–T1	9	2.75
C5–T1	75	22.94
Cord/cord branch (infraclavicular)	68	20.80
Supraclavicular and infraclavicular	17	5.20
Total	327	100.00

Neurolysis alone was performed in 152 patients, as all nerve elements remained continuous and capable of nerve action potential conduction. Direct end-to-end suturing was performed in 10 patients, including C5–C6 (*n* = 2), C5–C7 (*n* = 1), C7–T1 (*n* = 1), and median, radial or axillary nerve (*n* = 6). Nerve grafting alone was performed in 15 patients, including grafting from the superior trunk to its posterior and anterior divisions (*n* = 8), from C7 to the middle trunk (*n* = 2), and reconstruction of musculocutaneous, median, radial, and axillary nerves (*n* = 5).

Nerve transfer alone (*n* = 101) or combined with grafting (*n* = 42) were performed using C5 to the posterior division of the superior trunk (*n* = 13), superior trunk (*n* = 1), anterior and posterior divisions of the superior trunk (*n* = 1), or posterior cord (*n* = 1); C6 to the anterior division of the superior trunk (*n* = 14) or superior trunk (*n* = 3); ipsilateral C7 to the inferior trunk (*n* = 2), superior trunk (*n* = 7), or anterior or posterior divisions of the superior trunk (*n* = 2); C8 or T1 to the inferior trunk (*n* = 4) or anterior division of the inferior trunk (*n* = 1); accessory nerve to the suprascapular nerve (*n* = 104); partial ulnar nerve to the musculocutaneous nerve (*n* = 50); partial median nerve to the musculocutaneous nerve (*n* = 28); and intercostal nerve to the musculocutaneous nerve (*n* = 3), medial cord/median nerve (*n* = 2), ulnar nerve (*n* = 2), or radial or axillary nerve (*n* = 3). Additional transfers included the triceps long head branch of the radial nerve to the axillary nerve (*n* = 2) and the brachialis (BA) branch to the median nerve or anterior interosseous nerve (AIN, *n* = 7).

In patients with total RA, contralateral C7 nerve transfer was performed. Specifically, 41 patients underwent a two-stage procedure: in the first stage, a pedicled ulnar nerve was used to bridge the contralateral C7 to the ipsilateral side; in the second stage, it was coapted to the target nerve. Among these patients, contralateral C7 nerve was transferred to the superior and middle trunks in 26 patients and to the superior or inferior trunk in 15 patients. Nerve grafting was performed in 57 patients. Donor nerves harvested included the ulnar nerve (*n* = 41), superficial branch of the radial nerve (*n* = 26), medial cutaneous nerve of the forearm (*n* = 21), and sural nerve (*n* = 8); some patients required more than one donor nerve. Additionally, free functioning gracilis muscle graft was performed in three patients, and tendon transfer in 4 patients.

### Follow-up outcomes

The mean follow-up duration was 45.60 ± 18.37 months (range, 24–96 months). All 338 patients who received non-surgical management achieved Grade 3 or higher at final follow-up. Among the 327 surgical patients, recovery rates differed significantly according to the timing of surgery (*p* < 0.001). Patients who underwent surgery within 3 months achieved the highest recovery rate (78.5%), followed by those operated at 3–6 months (50.0%) and those beyond 6 months (45.2%).

Additionally, recovery rates differed significantly across injury patterns (*p* < 0.001, Table 6). Patients with C5–T1 injuries had significantly lower recovery rates than those with C5–C6 injuries (*p* < 0.001) or cord/branch injuries (*p* < 0.001). Regarding surgical procedures, overall recovery rates varied significantly among the four main procedural groups (*p* < 0.001), with the highest rate observed in the neurolysis-alone group (79.17%).

**Table 6:** Number of surgical patients recovering to Grade M3 or better after surgery (> 2 years of follow-up).

Injury patternSurgical procedure	No. of patients (%)					
	C5–C6	C5–C7	C5–T1	Cord/nerve	Supraclavicular and infraclavicular	Total
Neurolysis only	42 of 49	17 of 23	14 of 22	40 of 46	1 of 3	114 of 144
Nerve suture	4 of 7	2 of 4	0	8 of 11	1 of 1	15 of 23
Nerve grafting with or without neurotization	5 of 7	5 of 8	7 of 14	5 of 7	2 of 4	24 of 40
Nerve transfer alone	18 of 26	12 of 27	14 of 35	3 of 4	2 of 7	49 of 99
Tendon transfer	0	1 of 1	1 of 2	0	1 of 1	3 of 4
Free functioning gracilis	0	0	1 of 2	0	1 of 1	2 of 3
Total	69 of 89	37 of 63	37 of 75	56 of 68	8 of 17	

## Discussion

### Epidemiology

This study represents the second phase of a longitudinal multicenter cohort initiated in 2004 (9), covering the period from 2017 to 2022. Compared with reports from other regions (2, 10), the annual number of BPI cases treated at our center continued to increase over the study period. This may be attributed to the large regional population, our center’s role as a major referral hub for complex injuries, and a growing proportion of referrals from neighboring provinces (12.78%). Growing referral awareness among clinicians at primary hospitals has also helped more patients access standardized care.

The injury mechanism profile of BPI in the present cohort differs notably from that reported in our previous study (9). First, improved traffic regulation and safety awareness may have contributed to a reduction in traffic-related BPI, from 64.7% in the previous cohort (9) to 45.86% in the present cohort. Concurrently, e-bike accidents emerged as the main traffic mechanism (27.82%), surpassing motorcycles (13.23%). This transition is consistent with China’s rapid e-bike proliferation and with evidence that e-bike injuries disproportionately affect riders aged ≥40 years and female users (11–13). Second, falls from a height of less than 2 m, which accounted for 17.44% of cases, were the second most common mechanism. This proportion was similar to findings reported by Boyle et al. (7). In our region, low-energy mechanisms typically cause incomplete BPI, which is more likely to be missed during early clinical assessment due to associated injuries and a lack of specialized training among non-specialist clinicians. As peripheral nerve surgery has become more widely practiced in our region, earlier and more accurate recognition has become feasible.

These shifts in injury mechanisms were accompanied by notable changes in patient demographics. Compared with our previous cohort (9), the male-to-female ratio narrowed (2.59:1 vs. 7.10:1), mean age increased (41.65 vs. 29.04 years), and the proportion of patients aged 20–39 years declined (35.49% vs. 59.41%). These demographic characteristics also differed from those reported in other studies. Kandenwein et al. (14) reported a mean age of 26.8 years in a German cohort, in which motorcycle accidents accounted for 65% of cases. Zaidman et al. (2) reported a mean age of 32.5 years and male-to-female ratio of 5.4:1 in Canada.

### Clinical manifestations

The severity of BPI is largely determined by the magnitude of the traumatic force. Compared with our previous cohort, in which total BPI accounted for 42.66% (9), the present cohort showed a substantially lower proportion (22.94%) and higher rates of incomplete BPI (51.07%). This is similar to the finding of Faglioni et al. (15) that total BPI (46.1%) in Brazil was predominantly associated with firearm trauma. Jain et al. (16) reported that in India, RA was primarily associated with motorcycle accidents, with total BPI accounting for 48.02% of cases. In this cohort, C5–C6 and C5–C7 injury patterns predominated. This is consistent with the biomechanical principle that traction between the head and shoulder initially injures the upper nerve roots, with extension to C8–T1 occurring only under greater force (17, 18).

Fractures of the upper extremity and shoulder girdle were the most frequent concomitant injuries, consistent with other reports (10, 15). The lower incidence of vascular injuries, compared with Western series (2, 19), may reflect the lower-energy mechanisms prevalent in our population. Shoulder dislocation was present in 20.30% of patients. As Gutkowska et al. (20) noted, such cases rarely involve complete nerve transection or RA, and conservative treatment is often associated with favorable outcomes. Although the humerus (27.52%) was the most common fracture site, only 71.04% of these cases required surgical intervention. This result was partly attributable to greater tuberosity fractures associated with shoulder dislocation, most of which can be managed non-operatively. In contrast, surgery was recommended for clavicular fractures (98.82%) to facilitate early rehabilitation and promote neurological recovery of the affected limb. Additionally, concomitant spinal or lower extremity fractures indicated high-energy trauma mechanisms, the majority of which required surgery. Notably, BPI is missed in 34% of polytrauma patients due to altered consciousness (21), underscoring the need for auxiliary examinations to facilitate early identification.

MRI sensitivity for RA detection has been reported at 92.9% (22), though early T2-weighted imaging may yield false positives due to post-traumatic edema (23, 24). Emerging techniques including diffusion tensor imaging (DTI) and diffusion tensor tractography (DTT) have shown promise in improving the diagnostic accuracy of MRI for BPI, with reported sensitivity and specificity exceeding 85 and 98%, respectively (25, 26). High-frequency ultrasound enables real-time dynamic assessment of nerve morphology and adjacent structures, although its accuracy is operator-dependent (27). Moreover, visualization of C8–T1 roots is limited, with the detection rate for T1 roots being only 79.4% (28). Nerve conduction studies and electromyography (NCS/EMG) are sensitive for detecting axonal injury but cannot reliably identify denervation changes until 10–21 days after injury (29). The optimal diagnostic approach should therefore be individualized based on injury timing, clinical presentation, and available tools.

### Treatment strategy

The proportion of patients undergoing surgery within 3 months improved from 11.37% in our previous cohort (9) to 59.63% in the present series, suggesting a reduction in referral delays associated with improved initial diagnostic accuracy. A standardized 3-month course of non-surgical management is recommended before considering surgery to avoid unnecessary operations (30). In the early phase, non-surgical management involves pain control, neurotrophic support, and limb protection (31). In the recovery phase, rehabilitation focuses on maintaining joint range of motion, preventing adhesive capsulitis, enhancing muscle strength, and applying neuromuscular electrical stimulation (32). For the treatment of concomitant injuries, we advocate a comprehensive decision-making approach based on the patient’s clinical condition. In cases involving vital organ damage or multiple fractures, stabilization of vital signs should take priority, with fracture fixation and BP repair performed either in a single stage or in a staged manner thereafter. For hemodynamically stable patients, simultaneous fracture fixation and BP surgery should be pursued whenever feasible within the safety limits of long-duration anesthesia, thereby minimizing the morbidity associated with a second operative intervention.

When clear indications of nerve rupture or RA are present, timely surgery is recommended, with the procedure guided by intraoperative findings (30). Specifically, if all nerve elements are found to be in continuity with preserved nerve action potentials on intraoperative electrophysiological testing, neurolysis is preferred. In cases of traumatic neuroma with loss of conduction, tension-free neurorrhaphy following neuroma excision is performed. Nerve grafting is reserved for cases in which direct coaptation cannot be achieved, though functional outcomes deteriorate considerably when graft lengths exceed 10 cm (33, 34). Accordingly, nerve transfer is superior to nerve grafting for long-segment nerve defects (35).

Nerve transfer utilizes donor nerves derived from either intraplexal or extraplexal sources. When C5 avulsion results in shoulder abduction dysfunction, posterior transfer of the accessory nerve to the suprascapular nerve provides favorable long-term outcomes, with over 66% of patients achieving Grade 3 or greater shoulder abduction strength (36). In C5–C6 avulsion injuries where elbow flexion is affected, double fascicular transfer is the preferred approach (37). Acharya et al. (38) reported that after Oberlin II double fascicular nerve transfer, 88.9% of patients achieved active elbow flexion and forearm supination. The mean range of active elbow flexion was 113.9° and active supination was 67.8°. In C8–T1 injuries resulting in finger flexion dysfunction, potential donor nerves for transfers to the AIN include BA branch, brachioradialis (BR) branch, and the extensor carpi radialis brevis (ECRB) branch. Among these, the BA branch is the most frequently used due to its safety and effectiveness, with a recovery rate of 59% (39). The ECRB branch transfer, although technically more demanding, achieves superior functional recovery compared with other donors (39, 40). In contrast, the BR branch yields the poorest outcomes and should be considered only when other donor nerves are unavailable (41). When intraplexal donors are unavailable, extraplexal nerves serve as viable alternatives. Both the phrenic nerve and intercostal nerves are reliable motor donors for brachial plexus reconstruction. However, preoperative pulmonary function testing is necessary, given their distinct respiratory risks (42, 43). The contralateral C7 nerve, containing 17,000–40,000 mixed axons, represents an important donor option for total RA, enabling meaningful functional restoration without lasting donor-site morbidity (44).

Prolonged denervation beyond 12–18 months leads to irreversible motor endplate fibrosis and Schwann cell dysfunction, markedly limiting the potential for functional recovery (45). Furthermore, joint stiffness secondary to prolonged immobility, together with progressive intramuscular fibrosis and denervated muscle fiber degeneration, may further compromise the outcomes of delayed nerve repair (46). In such cases, functional reconstructive surgery is recommended. These procedures include tendon transfer, free functioning muscle transfer, and joint arthrodesis.

### Follow-up outcomes

All 338 non-surgical patients achieved Grade 3 or higher at final follow-up, consistent with the generally favorable natural history of incomplete or infraclavicular BPI managed conservatively (30). Among the surgical group, earlier intervention was associated with significantly better outcomes: patients operated within 3 months achieved a recovery rate of 78.5%, compared with 50.0% at 3–6 months and 45.2% beyond 6 months (*p* < 0.001). Clinical outcomes were significantly associated with injury extent and anatomical level: patients with infraclavicular injuries achieved the highest recovery rate (82.35%), followed by C5–C6 (77.53%), C5–C7 (58.73%), and C5–T1 (49.33%), consistent with benchmarks reported in the literature (14, 15, 17, 19, 47).

The lower recovery rate of nerve transfer (49.50%) relative to neurolysis (79.17%) and direct nerve repair (65.22%) reflects case selection bias rather than procedural inferiority, as nerve transfer is usually employed for severe BPI that cannot be directly repaired. The outcomes of C5–T1 injuries remain unsatisfactory (49.33%), even with contralateral C7 transfer in total RA patients. Therefore, earlier specialist referral and better reconstructive strategies are needed.

## Conclusion

This study demonstrates that the epidemiology of traumatic BPI in Guangxi, China, changed markedly over the study period from 2017 to 2022. E-bike accidents surpassed motorcycle accidents to become the leading mechanism of traffic-related BPI, coinciding with an aging patient population and a narrowing sex ratio. BPI was predominantly closed and incomplete, with the C5–C7 roots being the most commonly affected. Early diagnosis, timely intervention, and individualized surgery contributed to favorable outcomes. Nevertheless, functional outcomes following total RA remain suboptimal despite advances in surgical technique. Therefore, targeted preventive strategies and multidisciplinary management of BPI are needed. This study has limitations inherent to its retrospective design. To address these limitations, large-scale, multicenter prospective cohort studies are warranted to generate higher-quality evidence and further optimize the long-term outcomes of BPI.

## Data Availability

The raw data supporting the conclusions of this article will be made available by the authors, without undue reservation.
